# Advertisements and the Medical Press

**Published:** 1909-11-06

**Authors:** 


### Advertisements and the Medical Press.

There has recently been some discussion as to the proper attitude which a medical paper should maintain with regard to what may be called Professional Advertisements appearing upon its back pages. One view is that it is an ethical blunder for such a paper to allow in its editorial columns any expression of opinion in the way of notice of proprietary or other articles which are advertised in it. It is contended that such expressions of opinion are apt to be removed from their context by unscrupulous firms and flourished abroad in the lay Press as emanating from inspired professional quarters, and thus used to lead astray the public. On the other hand, there are those who submit that it is imperatively necessary for a medical paper to have the courage of its convictions, to tell its readers what it thinks without allowing itself to be influenced by what may be done with such expressions of opinion by advertisers.

Of these two views, the second appears to us to be more in consonance with the best ideals both of the medical profession and of journalism. A paper has obligations not only to its readers, but to its advertisers as well, and it best fulfils such obligations by being strictly honest and, as far as possible, impartial. There can be no extenuation whatever for the "puff professional," and no consideration of interest can justify the inclusion in editorial matter of paragraphs sent in by advertisers with the expressed wish that they should be taken up and inserted as the unbiassed opinion of the editorial investigator. On the other hand, there can be no excuse for not bringing to the notice of readers the merits of a new drug or the advantages of a new appliance even though such comment, made expressly in the interests of professional readers, be afterwards foisted upon an indiscriminating public. There is, it appears to us, greater need to interfere with the advertiser who distorts professional comment, as expressed in clinical lectures or in textbooks, to suit his own purposes, than to restrict the medical Press. At the same time it is worth while to point out that there has been a tendency in some quarters to regard "notices" in the editorial columns in too narrow a sense. Notices in a professional paper stand on a somewhat different footing from those appearing in the lay journals; they are presumably expert opinion, duly weighed and considered before being published. As such their compilation demands a scrupulous regard for exactness of phrase and honesty of import which should never be allowed to interfere with the commercial claims of the paper. Notices appearing in columns of every medical paper of any standing, in this country at least, are subject to editorial control, and we do not believe that there has been any abuse of the ordinary privileges which have been conceded to medical editors to comment on such notices.

